# Hypnosis as an Adjunct to Topical Anesthesia for Awake Intubation in a Patient With Difficult Airway: A Case Report

**DOI:** 10.1155/cria/9699223

**Published:** 2025-11-20

**Authors:** Stefan Torlakovic, Corinne Grandjean-Progin, Florence Nikles, Olivia Stiennon

**Affiliations:** ^1^ Department of Anesthesia, Cantonal Hospital of Fribourg (HFR), Fribourg, Switzerland, h-fr.ch; ^2^ Department of Otorhinolaryngology, Head and Neck Surgery, Cantonal Hospital of Fribourg (HFR), Fribourg, Switzerland, h-fr.ch

**Keywords:** awake intubation, fiber-optic intubation, hypnosis

## Abstract

**Background:**

Awake intubation is often distressing for patients, particularly in cases of difficult airway anatomy.

**Case Presentation:**

We report a case of successful awake fiber‐optic intubation in a 69‐year‐old male with limited mouth opening due to previous ENT surgeries. Hypnosis was used alongside topical anesthesia and low‐dose dexmedetomidine. The patient entered a relaxed state, and the intubation proceeded without pain, discomfort, or complications.

**Conclusion:**

This case demonstrates the feasibility of using hypnosis as a noninvasive adjunct to improve comfort during awake intubation. Further studies are warranted.

**Summary**

• Hypnosis can be an effective adjunct to topical anesthesia for awake intubation in patients with difficult airways.
• It may improve patient comfort, reduce anxiety, and facilitate cooperation during procedures.
• Successful implementation requires proper planning and training in medical hypnosis.
• This approach may reduce the need for pharmacological sedation in selected cases.

## 1. Background

Awake intubation is a technique used to manage anticipated difficult airways. Although effective, it is often perceived by patients as uncomfortable or even traumatic. Various protocols have been developed to perform awake intubation while maximizing patient comfort. At our institution, the standard approach involves local anesthesia combined with procedural sedation.

Several methods of airway anesthesia are described. Topical anesthesia can be administered using several devices, such as a mucosal atomization device, nebulizer, direct application, or via a translaryngeal block. Regional anesthesia may involve glossopharyngeal, superior laryngeal, and recurrent laryngeal nerve blocks. A combination of topical and regional techniques is commonly used. Sedation can be added to provide analgesia and anxiolysis, typically through intravenous infusion or boluses of agents such as remifentanil, dexmedetomidine, midazolam, or ketamine [1, 2].

Hypnosis is a waking state in which a person’s attention shifts away from the external environment and becomes focused on internal experiences such as thoughts, feelings, and imagery. Through focused attention and imagination, the patient may perceive imagined events as real. With the help of suggestions, the clinician and patient collaboratively create a hypnotic reality [3]. When used as an adjunct to standard anesthetic care, hypnosis has been associated with reduced perioperative pain and anxiety in selected patients, despite decreased administration of analgesic and sedative agents [4, 5]. Our institution recently began using hypnosis as a replacement of general anesthesia (for example, in breast surgery) or as an adjunct to regional anesthesia (for example, in thyroidectomy).

A literature search using the keywords “awake intubation,” “fiber‐optic intubation,” and “hypnosis” in Medline and Google Scholar did not find any articles describing the use of hypnosis for awake intubation.

This case report presents a patient who benefited from awake intubation using hypnosis and topical anesthesia.

## 2. Case Presentation

**Figure 1:**
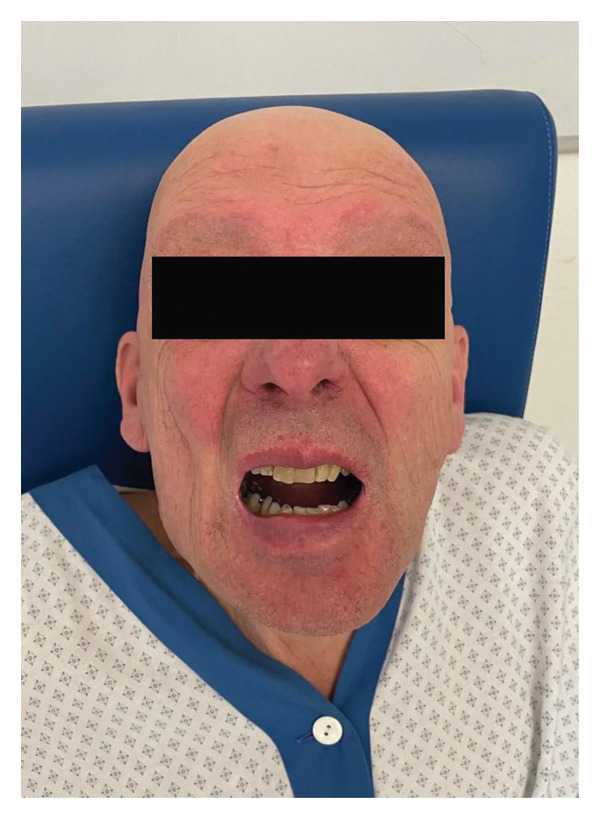
Maximal interincisal space.

The patient is a 69‐year‐old man (178 cm, 72 kg) with a complex ENT history, including facial trauma and oncological surgery for right superior gingival squamous cell carcinoma. He underwent right partial superficial maxillectomy with facial reconstruction in 2014 and adjuvant radio‐ and chemotherapy in 2015, complicated by a palatal fistula repaired in 2017 but with later recurrence. As a result of the multiple treatments, he presented a limited mouth opening (interincisal space of 1 cm) (see Figure 1: picture: the patient with maximal mouth opening [interincisal space of 1 cm]). He was scheduled for a panendoscopy following the discovery of a right nodal recurrence with infiltration of the masticator space.

During his previous surgery, he underwent an awake intubation under sedation with remifentanil and low dose propofol. He experienced it as painful and distressing, which left him apprehensive about the upcoming procedure.

### 2.1. Investigations

No specific investigations were required beyond the clinical evaluation for anticipated difficult airway due to known anatomical limitations. A standard preoperative assessment was conducted.

### 2.2. Differential Diagnosis

Not applicable in this context. The indication for awake intubation was based on known anatomical abnormalities and previous surgical history.

### 2.3. Treatment

During the preanesthesia consultation, hypnosis was proposed as a complementary technique to standard topical anesthesia for awake intubation. This approach had not previously been used by the anesthesiologist for this specific indication nor had it been done in the institution.

The hypnosis anesthesia fellow in charge of the patient is also a formally trained hypnotherapist; she completed training in medical hypnosis, including a 12‐day theoretical and practical course focused on acute pain management and procedural analgesia. The training was delivered by the French Institute Emergences [6], which is recognized by the French National Agency for Continuing Professional Development (Agence nationale du Développement Professionnel Continu).

A multidisciplinary briefing was held with the ENT surgeon and the anesthesia team to plan the procedure. Awake intubation under a hypnosis, combined with topical anesthesia and a low‐dose infusion of dexmedetomidine, was planned. In the case of failure, the procedure would be postponed. In the case of an emergency, the ENT surgeon would be in the operating room to perform a front of neck access.

On the day of the operation, two medical teams were present. The first team comprising an anesthesiology nurse, a resident, and an attending was responsible for the local anesthesia and performed the awake intubation. The second team included the anesthesia fellow who had performed the preanesthesia consultation was responsible for the hypnosis. She visited the patient in his hospital room before the operation. During a 20‐min session, the patient shared a positive meaningful memory and described it in details. An aerosol of 2% lidocaine (5 mL) was administered during this session.

The hypnotherapist escorted the patient to the operating room. Peripheral venous access and standard monitoring were applied to the patient (ECG, NBIP, and SpO2). Using a mucosal atomization device, lidocaine 10% was applied to the left nostril and the oropharynx.

The anesthesiologist induced the hypnosis using the Ericksonian technique. The patient was invited to focus on a point with his eyes, then to concentrate on his breathing, and finally to relive the positive memory he had chosen and described earlier. The hypnotherapist described the place, the colors, and the smells, using a calm and monotonous voice. The therapist spoke throughout the entire procedure, employing techniques to dissociate the patient from the operating room.

The patient entered a state of well‐being, and the technical procedure began. A continuous infusion of dexmedetomidine, an α2‐agonist, was started at 0.4 mcg/kg/hour (a dose lower than the one used for sedation). The patient exhibited no reaction to the insertion of the endotracheal tube through the left nostril or to its progression to the vocal cords. Intubation was performed using a reusable fiber‐optic scope. The procedure was technically easy, and the patient did not cough.

Once the endotracheal tube was correctly positioned, general anesthesia was induced with propofol and fentanyl and subsequently maintained with a continuous infusion of propofol and remifentanil. The surgery, a panendoscopy, lasted 42 min, with a total anesthesia time of 63 min.

### 2.4. Outcome and Follow‐Up

The patient emerged from anesthesia without pain or disorientation. He reported a VAS pain score of 1/10 and was discharged from the recovery room after 46 min. He expressed satisfaction and no distress regarding the intubation experience. The pain during the intubation was evaluated with a VAS of 5/10. Concerning anxiety, the patient evaluated it at a VAS of 7/10 before intubation, 3/10 during intubation, and 0/10 in recovery room. No complications were observed.

## 3. Discussion

The hypnosis is increasingly recognized for its potential to improve patient experience in surgical settings. Studies have shown benefits in breast surgery and thyroidectomy, particularly regarding patient satisfaction, anxiety reduction, and pain control [5, 7].

This case illustrates that the hypnosis, when performed by a trained clinician, can serve as a valuable adjunct to topical anesthesia in awake intubation. It may be especially helpful in patients with difficult airway anatomy who are apprehensive due to prior negative experiences.

Limitations include the need for trained hypnotherapists and the lack of broader clinical validation. Nevertheless, this case supports further investigation into the hypnosis as a nonpharmacological aid in airway management.

## Consent

Patient consent was obtained.

## Patient Perspective

“I had a very bad experience during my last surgery with awake intubation. This time, the hypnosis made everything different. If I ever need again an anesthesia I would like the same approach. I’m very grateful.”

## Conflicts of Interest

The authors declare no conflicts of interest.

## Funding

No funding was received for this manuscript.
